# Influence of the Silver Content on Mechanical Properties of Ti-Cu-Ag Thin Films

**DOI:** 10.3390/nano11020435

**Published:** 2021-02-09

**Authors:** Saqib Rashid, Marco Sebastiani, Muhammad Zeeshan Mughal, Rostislav Daniel, Edoardo Bemporad

**Affiliations:** 1 Engineering Department, University of Rome “Roma Tre”, via della Vasca Navale 79, 00146 Rome, Italy; marco.sebastiani@uniroma3.it (M.S.); zeeshan.mughal@open.ac.uk (M.Z.M.); edoardo.bemporad@uniroma3.it (E.B.); 2 School of Engineering & Innovation, The Open University, Walton Hall, Milton Keynes MK7 6AA, UK; 3 Department of Materials Science, University of Leoben, Franz-Josef-Straße 18, 8700 Leoben, Austria; rostislav.daniel@unileoben.ac.at

**Keywords:** Ti-Cu-Ag thin films, mechanical properties, magnetron sputtering, nanoindentation, FIB-DIC

## Abstract

In this work, the ternary titanium, copper, and silver (Ti-Cu-Ag) system is investigated as a potential candidate for the production of mechanically robust biomedical thin films. The coatings are produced by physical vapor deposition—magnetron sputtering (MS-PVD). The composite thin films are deposited on a silicon (100) substrate. The ratio between Ti and Cu was approximately kept one, with the variation of the Ag content between 10 and 35 at.%, while the power on the targets is changed during each deposition to get the desired Ag content. Thin film characterization is performed by X-ray diffraction (XRD), nanoindentation (modulus and hardness), to quantitatively evaluate the scratch adhesion, and atomic force microscopy to determine the surface topography. The residual stresses are measured by focused ion beam and digital image correlation method (FIB-DIC). The produced Ti-Cu-Ag thin films appear to be smooth, uniformly thick, and exhibit amorphous structure for the Ag contents lower than 25 at.%, with a transition to partially crystalline structure for higher Ag concentrations. The Ti-Cu control film shows higher values of 124.5 GPa and 7.85 GPa for modulus and hardness, respectively. There is a clear trend of continuous decrease in the modulus and hardness with the increase of Ag content, as lowest value of 105.5 GPa and 6 GPa for 35 at.% Ag containing thin films. In particular, a transition from the compressive (−36.5 MPa) to tensile residual stresses between 229 MPa and 288 MPa are observed with an increasing Ag content. The obtained results suggest that the Ag concentration should not exceed 25 at.%, in order to avoid an excessive reduction of the modulus and hardness with maintaining (at the same time) the potential for an increase of the antibacterial properties. In summary, Ti-Cu-Ag thin films shows characteristic mechanical properties that can be used to improve the properties of biomedical implants such as Ti-alloys and stainless steel.

## 1. Introduction

Over the years, metals have been widely used for biomedical implants because of their properties, such as mechanical strength, corrosion resistance, and biocompatibility [1,2]. Several metallic elements and their alloys such as titanium (Ti), Ti-based alloys, platinum (Pt), and austenitic stainless steel (316 L) are used for orthopedic and biomedical applications [3]. However, degradation of metallic implant takes place due to the interaction with corrosive media inside the human body with a subsequent release of metallic ions, such as nickel (Ni) ions released from corrosion of titanium-nickel (Ti-Ni) alloy implants, which is harmful for the patients [4,5]. To solve the metallic degradation problem, surface treatment or application of thin films are common techniques used to improve the chemical, mechanical, and biological properties of the surface interfaced with the living tissues [6]. For biomedical implants, the most important factor is to have excellent corrosion resistance and low toxicity due to released metallic ions [7], possibly in combination with antibacterial or even antiviral functionalities [8,9].

Mostly monolayers or multilayers of oxides, nitrides, and carbides thin films of different elements such as Zr, Ti, Cu, Ni, Au, and Ag are used in order to enhance the surface mechanical properties [10]. However, in order to further improve the corrosion resistance and biocompatibility of the implants, the implant alloys are often coated with various multi-element thin films such as Zr-based thin films (Zr-Cu, Zr-Cu-Ag, ZrCN, Zr/ZrCN multilayer) [11,12,13] and Ti-based thin films (TiN, Ti-Cu, Ti-Zr-Si) [14,15]. The Young’s modulus and hardness of Zr-based thin films were reported in the range of 95–121.7 GPa and 5–7 GPa, respectively [12,16]. Ti-based thin films showed high hardness in the range of 5–12.5 GPa and elastic modulus of 90–200 GPa [17,18]. Metallic thin films have recently emerged as alternative advanced surfaces for many applications, such as micro/nano-electromechanical systems and for biomedical use. For instance, due to their structural features and physiochemical properties, interest is rising for metallic thin films in the field of bio-implants and surgical tools [19].

In particular, Ti-based intermetallic thin films could combine promising biocompatibility and high durability [18,20]. Among several available techniques, physical vapor deposition (PVD), e.g., sputtering, is one of the most commonly used techniques to coat implants [21], as the films produced by the sputtering process have very low surface roughness, which also play an important role on biofilm adhesion on Ti-based thin films [22,23,24]. The addition of several metallic elements during processing can improve the properties of pure Ti by the formation of intermetallic compounds. Specifically, silver (Ag) and copper (Cu) add new properties without showing a significant difference in biocompatibility [25]. The properties of intermetallic thin films are usually better than pure metallic films due to their mixed bonding (metallic, covalent, and ionic) at specific stoichiometry [26]. Many authors have studied the antibacterial behavior of metallic elements such as Au, Cu, Zn, Ag, and their alloys because of their ability to kill bacteria up to a broad spectrum [27]. The antibacterial mechanism of silver is complicated due to its interaction with thiol groups of bacteria proteins which may affect the replication of DNA [28]. However, various forms of Ag have been used in medicine in the past, including pure metal [29], silver nitrate [30], silver salts [29], silver polymer composite [8], silver nanoparticles [29,31], and Ag-based thin films [32].

In this framework, the study of the influence of Ag addition on the properties of Ti-based alloys still represents a major challenge in the scientific community. In fact, the improvement of antibacterial properties is usually accompanied by an undesired decay of the mechanical and tribological behavior, which needs to be solved. Although Ag has been used with Zr-based thin films such as Zr-Cu-Ag in previous studies [33,34], to the best of the authors’ knowledge, Ti-Cu-Ag thin films have never been used to coat biomedical implants, although there has been an increasing interest in application of Ti-Ag-based thin film systems in the biomedical field because of the two-fold advantage of this system: biocompatibility of titanium and antimicrobial effect of silver [32,35]. Copper is considered a promising element in the field of biomedical applications because of its antibacterial activity against numerous bacteria [36]. The biocidal performance of copper is associated to liberation of Cu^+1^ and Cu^+2^ ions [37,38,39] as observed in Ti-Cu [40].

In recent years, copper-based systems have also been proposed for the production of surface antiviral coatings with virucidal properties, especially in view of the recent COVID-19 pandemic [41,42]. Very recent examples include [42,43] copper-coated touch surface fabricated by cold-spray technology, as well as antiviral Cu*_x_*O/TiO_2_ photo catalyst thin films with interesting photo-activated anti-viral properties.

These examples from the literature suggest the Ti-Cu-Ag system to be a promising ternary system for the production of surface with potential multiple biocidal functionality (both antibacterial and antiviral). At the same time, the analysis of feasibility for the real application of this ternary system requires a fundamental study on structure –microstructure –property correlation of the films, with specific focus on the mechanical properties that are a fundamental requirement for real industrial applications.

For this reason, in the present study we have produced and characterized Ti-Cu-Ag thin films with a varying Ag content from 10 to 35 at.% on Si substrates by co-deposition of Ti, Cu, and Ag and studied thin films. The effect of the Ag content in the Ti-Cu-Ag composite thin films on their microstructure and mechanical properties is evaluated. Although the biocompatibility and antimicrobial and antibacterial properties are commonly the primary properties of interest of thin films for biomedical applications, we first focused on the correlation between the thin film composition and microstructure and their mechanical properties, which are as decisive for the application of thin films for implants as their antibacterial properties.

## 2. Materials and Methods

### 2.1. Thin Film Deposition

**Figure 1:**
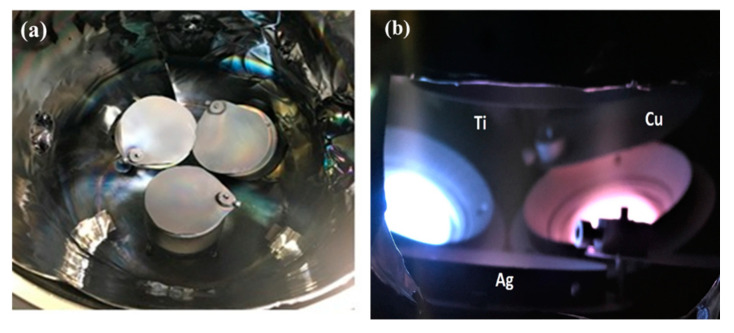
(**a**) Physical vapor deposition (PVD) chamber of three tilted metallic targets with respect to the substrate holder, and (**b**) targets ignite during sputtering.

A series of Ti-Cu-Ag thin films was deposited on rotating 525 μm-thick silicon (100) substrates (21 × 7 mm^2^) by direct current (DC) magnetron sputtering in a custom-made deposition system equipped with three unbalanced magnetrons. Three pure metallic targets, Ti, Cu, and Ag (3″ in diameter), were employed with confocal arrangement at a maximum tilting angle of ~50 degrees with respect to the substrate normal (see Figure 1). The composite monolayer thin films were produced by the co-sputtering of all three targets at the same time at room temperature without applying negative substrate bias voltage. The power supplied to metallic targets to obtain the desired film compositions is summarized in Table 1. All the substrates were cleaned in ultrasonic bath and ethanol for 10 min before mounting on the substrate holder at equidistant positions with a Kapton polyimide tape. The distance between the substrates and targets was 70 mm. The substrate rotation was kept constant at 80 rpm for all thin films.

The deposition chamber was pumped down to a vacuum of approximately 1 × 10^−5^ Pa before each deposition run. The argon pressure was maintained at 0.52 Pa corresponding to the gas flow of 30 sccm for all thin films. For further substrate cleaning and surface activation, a preliminary etching step was performed, powered by radio frequency (RF) power supply at 50 KHz, at an argon pressure of 1.2 Pa and a discharge power of 0.03 KW. During the deposition, DC discharge power was applied to the magnetrons in a power-controlled mode for 40 min (see Table 1), resulting in film thickness varied between 1.40 and 1.48 µm, depending on the film composition.

### 2.2. Characterization of Thin Films

The crystallographic structure of the thin films was investigated by X-ray diffraction (XRD) using a θ-2θ Bruker D8 Advanced system with Cu Kα radiation (λ = 0.154 nm). Diffraction patterns were collected by using grazing incident angle of 1 degree with time step of 0.02°/s. The operating voltage and current were 40 kV and 40 mA, respectively. The elemental composition of the thin films was evaluated via energy dispersive X-ray spectroscopy (EDX, Oxford Instrument INCA, High Wycombe, UK), using built-in sensitivity factors for calibration.

The film thicknesses were measured by using a white light optical profilometer with a Leica DCM 3D software package via automatic step measurement of coated and uncoated parts of the substrate. All the deposition rates were calculated as film thickness over the time, neglecting eventual differences in the film density. The surface topography and average roughness R_a_ of the thin films were analyzed and measured using a Bruker atomic force microscope (AFM Dimension Icon, Bruker^®^, Billerica, MA, USA) system scanning in a tapping mode with high aspect ratio tips according to the method described in our previous paper [44]. The tips used have half-apex angle of <25° with a spring constant of 42 N/m and a resonant frequency of 320 KHz. All measurements were taken on an area of 5 × 5 µm^2^ and the data was processed using a Bruker Nanoscope Analysis software v1.40r1 suite.

Elastic modulus (E) and hardness (H), were determined using nano-indentation testing method, using a KLA-Nanomechanics G200, (KLA Corporation^®^, Milpitas, CA, USA), fitted with a Berkovich diamond indenter operated in continuous stiffness measurement mode, hence allowing obtaining both **E** and **H**, as a continuous function of the depth from a single indentation experiment [45]. A standard fused silica sample was tested before and after a batch of measurements to calibrate the tip and ensure the reliability of the results. For all measurements reported here, a minimum of 25 indentations were performed and the calculations were made by the Oliver and Pharr method from the load-displacement curve using 10% of the film thickness at the maximum indentation depth [46].

For adhesion evaluation, scratch tests were performed using an Anton Paar Revetest Xpress micro scratch tester (Anton Paar^®^, Graz, Austria) in which critical load acting normal to the thin film surface at incident of failure is related to adhesion between thin film and substrate. However, critical load depends on mechanical strength (adhesion) of the thin film-substrate system but also on various parameters such as scratch indenter tip radius, film thickness, friction effects, hardness, and elastic modulus of coatings and substrate material. During the test, scratches were made on the surfaces with a sphero-conical diamond tip (200 µm radius) which is drawn at a constant speed (16.5 mm/min) under a progressive load (1–30 N) with a fixed loading rate (120 N/min).

### 2.3. Residual Stress Measurements

**Figure 2:**
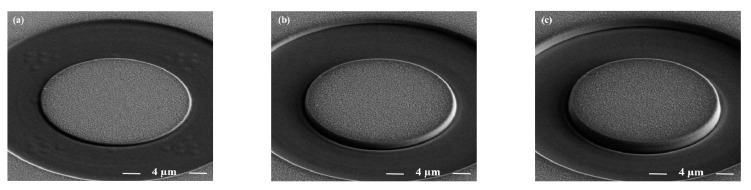

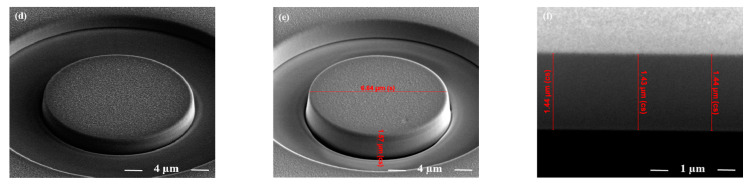
Representative images from (**a**–**e**) step-by-step milled pillar showing the semi-automatic milling procedure (scale bar only in figure (**e**)), and cross section (**f**), highlighting the thin film thickness.

The residual stress measurements were carried out by the focused ion beam and digital image correlation (FIB-DIC) micro-ring core method on a FEI Helios Nanolab 600 dual beam focused ion beam scanning electron microscope (FIB/SEM, Thermo Fisher Scientific, Waltham, MA, USA), using a specifically developed automated procedure [47]. The milling was performed using an annular trench with an inner diameter of 6.5 μm while employing a current of 0.92 nA at the acceleration voltage of 30 kV. Ten high resolution secondary electron images were acquired before and after each milling step using an integral of 128 images at a dwell time of 50 ns. The automatic procedure continuously monitored and corrected electron and ion beam drift while maintaining the same contrast of the reference image. The milling was performed until the h/D ratio of 0.2 was achieved, where h and D represent the milling depth and the pillar diameter, respectively. The h/D ratio of 0.2 ensures an optimal strain relief, as demonstrated in number of recent publications [48,49,50]. After the milling cycle, all images were processed with a customized MATLAB v2.1.0.0 based DIC code [51] to calculate the relaxation strain over the pillar. Assuming an equi-biaxial stress distribution in the thin film, which is more than reasonable in the case of PVD films on flat substrates, average stress in the film was calculated by using the interpolated relaxation strain at h/D = 0.2, elastic modulus (E), and Poisson’s ratio (ν) according to the following equation [52]:

(1) $$\sigma =\;-\frac{E\Delta \varepsilon }{\left(1-\nu \right)}$$

where, *σ* is the average residual stress in the film and Δε is the relaxation strain. Thin film thickness was also measured by using the FIB cross-section at a current of 0.92 nA at 30 kV. The step-by-step milling procedure highlighting the different stages of milling process along with the thin film cross-section is represented in Figure 2.

The residual stress state was also indirectly determined by substrate curvature measurements, according to the Stoney’s equation:

(2) $$\sigma_{f}=\left\{\frac{M_{s}}{6\left(1-v_{s}\right)}\right\}\left(\frac{t_{s}^{2}}{t_{f}}\right)\left(\frac{1}{R}-\frac{1}{R_{0}}\right)$$

where $\sigma_{f}$ is the residual stress in the thin film, $t_{f}$ the film thickness, $t_{s}$ the substrate thickness, $M_{s}$ the biaxial elastic modulus of the substrate (180 GPa, in the present case), $v_{s}$ is the Poisson’s ratio of the substrate, $R_{0}$ and R are the radii of curvature of the substrate before and after the thin film deposition, respectively.

## 3. Results and Discussion

### 3.1. Elemental and Structural Characterization

The Ti-Cu-Ag composite thin films with a varying Ag content were grown under optimized process conditions with regards to the target power and working pressure, while the Ti/Cu ratio in the thin films was kept approximately equal to 1 by a slight variation of the target power, the Ag content was varied between 10 and 35 at.% by a progressive increase of power on the Ag target. The exact elemental composition from EDX measurements is shown in Table 1.

**Figure 3:**
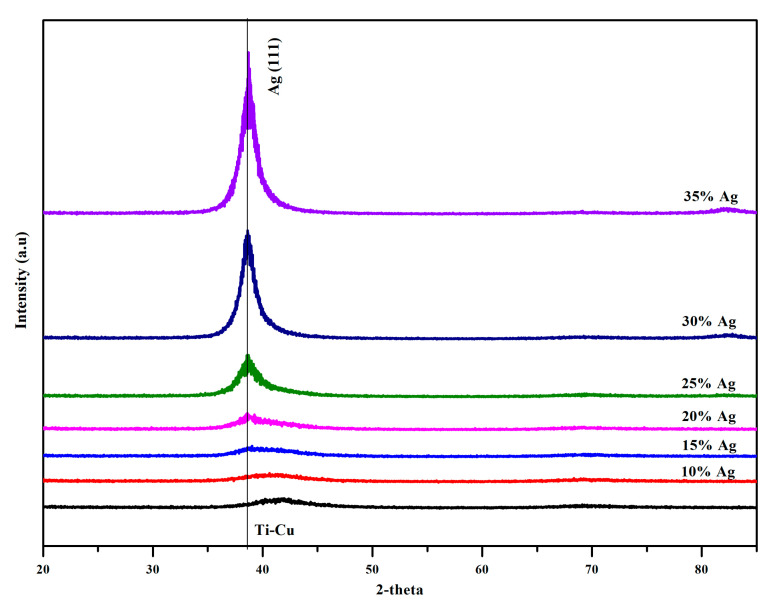
X-ray diffraction patterns of the Ti-Cu and Ti-Cu-Ag composite thin films on a silicon substrate with an Ag content varying between 10 and 35 at.%.

X-ray diffraction patterns of the as-deposited thin films are shown in Figure 3. For the binary Ti-Cu thin films, only a broad diffraction hump in the [38,39,40,41,42,43,44,45] 2θ-range is observed without any pronounced Bragg’s diffraction peaks which represent amorphous glassy structure with Ti-Cu ratio nearly equal to one. Although the Ti-Cu alloys form multiple intermetallic compounds, a solid solution forms for alloys with the Ti_48_Cu_52_ composition [32]. This was the target composition of the Ti-Cu thin film in this study in order to get the amorphous bulk metallic glass structure [34]. While keeping the Ti/Cu ratio constant and increasing the Ag content, the diffraction hump shifted towards lower 2θ angle. The position of the maximum level of broad peak can be correlated, in the first approximation, to the typical distance between nearest-neighbor atoms within the amorphous matrix. The decrease in the nearest neighbor distance with Ag content can be associated with the progressive substitution of Ti atoms by Cu and Ag atoms.

While the structure of the Ti-Cu-Ag composite thin films with the Ag content lower than 25 at.% remains amorphous similar to the binary Ti-Cu thin film (Figure 3), a more pronounced diffraction peak was observed for the ternary Ti-Cu-Ag thin films with the Ag content varying from 25 to 35 at.% reflecting their nanocrystalline structure. The full width at half maximum (FWHM) of the diffraction peak is obviously higher for the binary Ti-Cu thin films than for the composite Ti-Cu-Ag thin films and decreased when the Ag content increased. This feature may reflect a more ordered structure of the Ti-Cu-Ag thin films than that of the Ti-Cu thin films especially for the Ag contents higher than 25 at.%. Therefore, the Ag content of ~25 at.% was identified as a critical threshold for the transition from an amorphous to a crystalline structure of Ti-Cu-Ag system.

### 3.2. Surface Morphology

**Figure 4:**
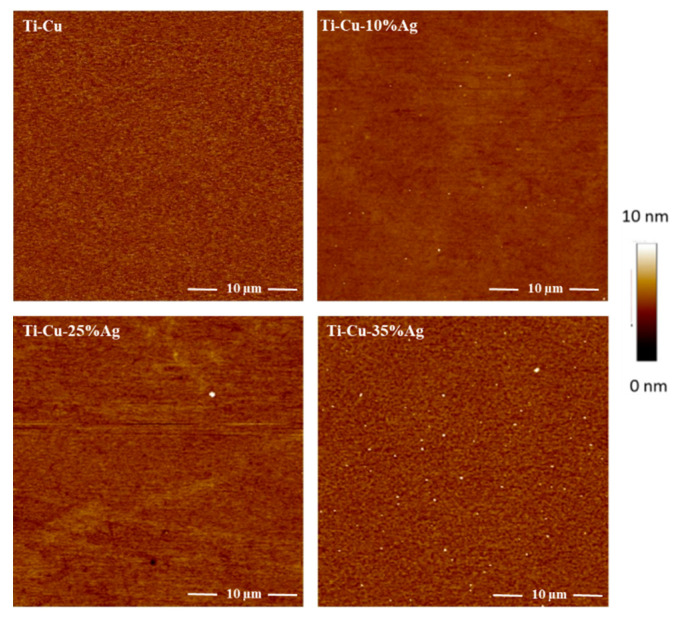
Atomic force microscope (AFM) images of the Ti-Cu and Ti-Cu-Ag thin films with 10, 25, and 35 at.% of Ag.

The surface average roughness R_a_ of the Ti-Cu-Ag thin films with a varying Ag content determined by AFM is shown in Figure 4. The AFM images reveal crack-free smooth surfaces for all thin films with an increase in roughness as the Ag content increases from 10 to 35 at.%.

**Figure 5:**
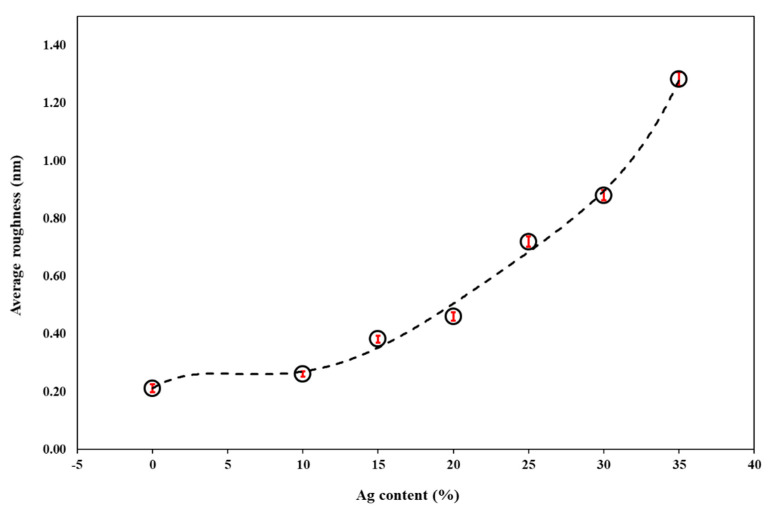
The average surface roughness of the Ti-Cu and Ti-Cu-Ag thin films as a function of the Ag content.

The surface roughness increase from 0.5 to 1.3 nm corresponds to the appearance of the crystalline phase at the Ag content of ~20 at.% and grain coarsening at higher Ag content of 35 at.% (Figure 4). On the other hand, the amorphous Ti-Cu thin film and Ti-Cu-Ag thin films with the Ag content below 20 at.% exhibit the average surface roughness well below 0.5 nm (Figure 5). Thin films with such a low average surface roughness are in general very favorable for antibacterial biomedical applications [8], especially if an adequate amount of antimicrobial agents such as Ag and Cu is added into the protective thin films, which induce a release of metallic ions after exposure to a humid environment [53].

### 3.3. Mechanical Properties

**Figure 6:**
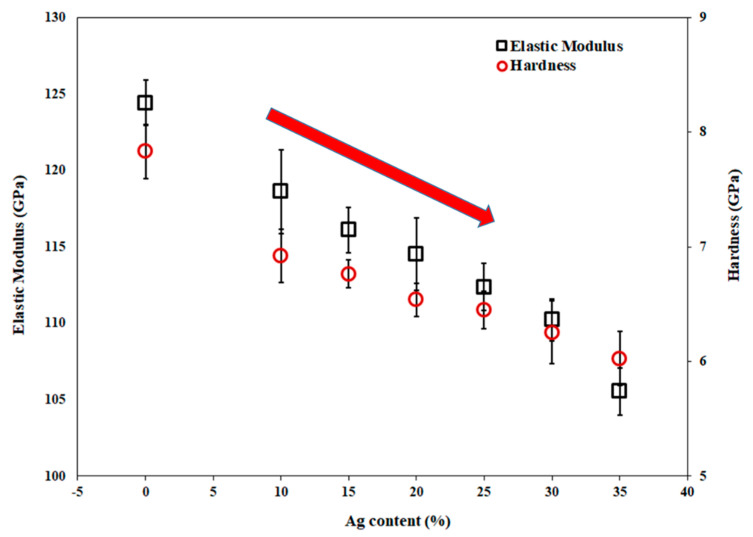
Elastic modulus and hardness of the Ti-Cu and Ti-Cu-Ag thin films as a function of the Ag content.

Thin films for the biomedical applications must have a high mechanical stability to insure their durability in service. The elastic modulus (E) and hardness (H) of Ti-Cu-Ag thin films as a function of the Ag content are shown in Figure 6. The highest E and H values of 124.50 GPa and 7.85 GPa, respectively, exhibit the Ag free Ti-Cu thin film. There is a slight decrease in both modulus (118.6 GPa) and hardness (7 GPa) with the addition of silver into the Ti-Cu thin films. With the first addition of 10 at.% Ag, it shows 5% decrease in modulus and 10% decrease in hardness. The phase changes from amorphous to partially crystalline at 25 at.% Ag and changes to completely crystalline with 35 at.% Ag, which causes the decrease in mechanical properties. At threshold phase change point of 25 at.% Ag, there is a decrease of 10% in modulus and 18% in hardness which is almost two times lower than controlled sample. With the further addition of Ag into the Ti-Cu thin films, both modulus and hardness continuously decrease to the lowest values of 105.5 GPa and 6 GPa for E and H, respectively, at 35 at.% of Ag in the Ti-Cu-Ag thin films. The percentage decrease in modulus is 15.6% and hardness is 23.5% for 35 at.% Ag as compared to the controlled sample. Although the nanocrystalline materials are commonly harder and stiffer due to grain boundary-related hardening [54,55], the decrease of E and H of the Ti-Cu-Ag thin films is dominated by the effect of the addition of soft Ag atoms into the thin films and may even be intensified by softening due to rotation and/or sliding of crystallites with sub-critical size [56,57]. 

### 3.4. Scratch Test

**Figure 7:**
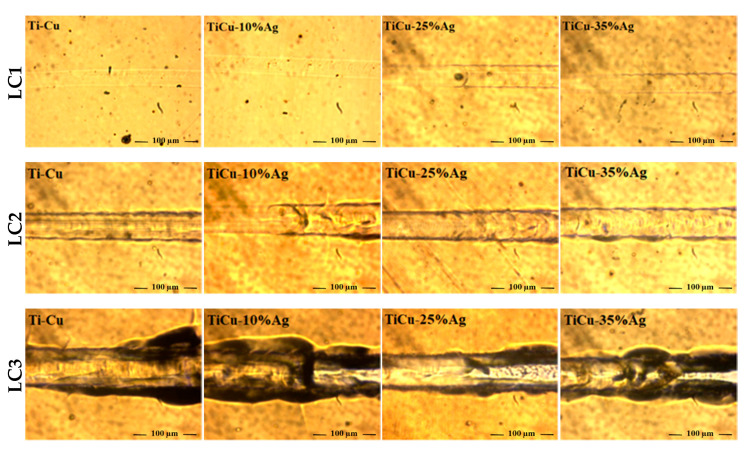
Representation of first failure LC1, second failure LC2, and complete delamination LC3 for the TiCu and TiCuAg thin films (scratch direction from left to right).

The scratches on the thin film surfaces were observed with an optical profilometer and images at incident of the first failure (LC1) are shown in Figure 7. The first failure in all the thin films was semi-circular rings due to buckling inside the scratch track. The chipping of the films (LC2) shows the first delamination in the scratch track. Finally, the complete delamination of the thin film from the substrate occurred and is represented in the images as failure at LC3.

**Figure 8:**
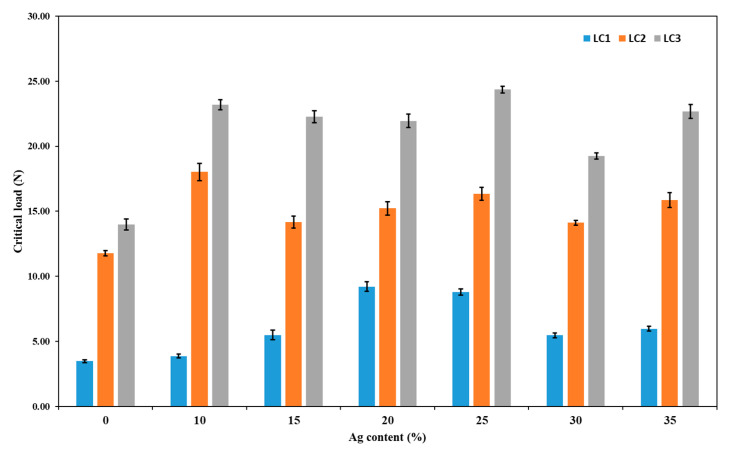
Critical load (LC1, LC2, and LC3) as a function of Ag content in the TiCu thin films.

Scratch adhesion test confirms that produced thin films have good adhesion with the substrate. All the critical loads as a function of silver content in the Ti-Cu thin films are represented in the Figure 8. The critical load LC1 increases with the significant increase in silver content up to 20–25 at.% and decreases for higher silver content. The silver containing thin films show significantly higher LC2 and LC3 values as compared to TiCu. The complete delamination of the thin films at higher critical load (20–25 N) reflects good adhesion with the substrate. The increase of critical load for increasing silver content is probably associated to the increase of ductility associated to the reduction of hardness for higher silver content [58,59].

### 3.5. Residual Stress Measurement

**Figure 9:**
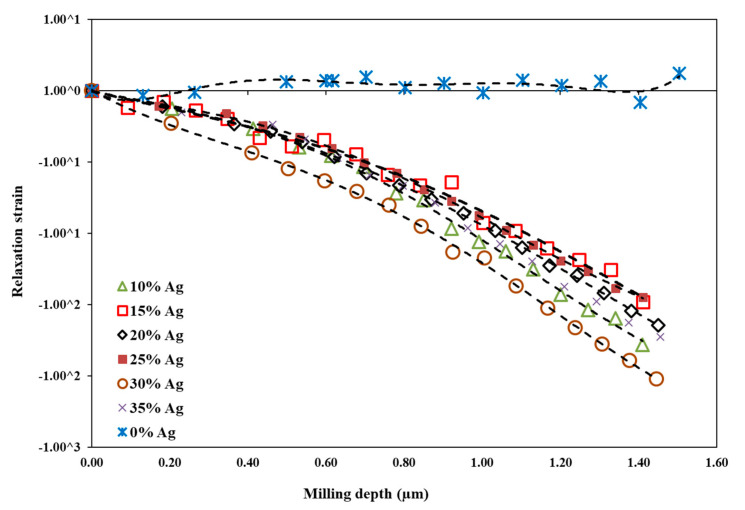
The relaxation strain determined by the focused ion beam and digital image correlation (FIB-DIC) method for Ti-Cu and Ti-Cu-Ag thin films with a various Ag content as a function of the milling depth.

The relaxation strain for the Ti-Cu and Ti-Cu-Ag thin films determined by the FIB-DIC method is shown in Figure 9. It represents a measure of the variation of the residual stress state induced during processing and as a consequence of the thermal expansion mismatch of a thin film and a substrate across the thin film thickness. The relaxation strain for the Ti-Cu thin films shows positive and constant values throughout the entire thin film thickness, which corresponds to a steady compressive residual stress state in the thin films. All the Ag-containing Ti-Cu-Ag thin films show negative relaxation strain values increasing from the thin film surfaces towards the thin film/substrate interface, which reflects a gradient in the tensile residual stress in the thin films. The development of the tensile stress state is obviously an effect related to the Ag addition, which correlates well with the decrease of hardness as the tensile residual stress in the thin films, together with the presence of Ag atoms, allows for more pronounced plastic deformation during nanoindentation.

Besides the absolute values, the slope of individual curves also gives us information about stress development in the thin films. The relaxation strain of the Ti-Cu-Ag thin film with 30 at.% of Ag reveals a larger slope of the curve compared to other Ti-Cu-Ag thin films, which indicates a greater residual stress gradient in the thin film, which is likely associated with the polycrystalline nature of the thin film microstructure. In fact, larger tensile stress developed in the thin films deposited under identical process conditions but different crystallite sizes is given by the interaction of surface atoms across individual growing islands in the initial growth stages of polycrystalline materials and reduction of the surface area when adjacent growing islands form the grain boundaries [60]. Another important contribution to the tensile stress, which may be more significant than grain boundaries effects, is an increasing thermal expansion mismatch strain between the high Ag-containing thin films and the Si substrate, especially for the Ti-Cu-Ag thin film with the Ag content higher than 25 at.% [61].

**Figure 10:**
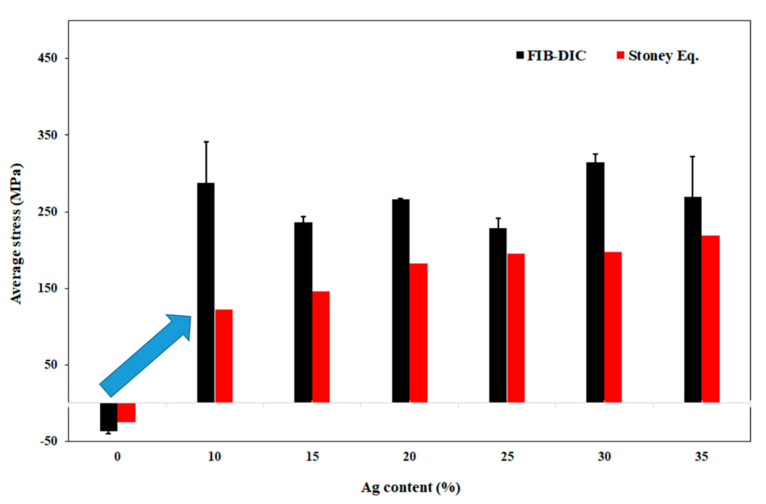
Average residual stress determined by the FIB-DIC method and from the substrate curvature measurements according to the Stoney’s equation as a function of the Ag content in the Ti-Cu-Ag thin films.

A comparison of average residual stress determined by using the FIB-DIC method and from the substrate curvature measurements is given in Figure 10. The residual stress obtained from both techniques shows a similar trend, with a clear transition from compressive to tensile stress in the thin films after the addition of silver. The Ti-Cu thin film exhibits compressive residual stress of −36.5 MPa and tensile stresses ranging from 228.5–314 MPa developed in the thin films after the addition of silver from 10–35 at.%. It is noteworthy that the values calculated by FIB-DIC are roughly 35% higher than the values obtained by the substrate curvature method according to the Stoney’s equation. This difference in the stress values determined from those two methods was already observed in our recent work and related to the difference of the representative volume element (RVE) in the two cases [48]. The curvature method takes into consideration the whole volume of the sample which can also include the stress relaxation effects associated with the microdefects and interfaces. On the other hand, FIB-DIC measures only a few cubic micrometers of the material that does not consider relaxation effects related to micro-cracks. Another important point to consider is that the wafer curvature method only takes into consideration the biaxial elastic modulus of the substrate, while the FIB-DIC method uses both the elastic modulus and the Poisson’s ratio of the thin film and substrate for calculating the residual stress.

In summary, the microstructural and nanomechanical characterization activities show a remarkable effect of silver addition on Ti-Cu-Ag co-sputtered thin films, where a transition from amorphous to crystalline and a more significant decrease of the mechanical properties are observed for Ag addition above 25 at.%.

The present study, therefore, suggests that Ti_35.5_-Cu_39.5_-Ag_25_ thin film, with increased adhesion, sufficiently good hardness, and partially crystalline structure, can be considered as the optimal composition out of produced thin films. The proposed configuration, therefore, could be a suitable candidate for the evaluation of biocompatibility, antibacterial, and antiviral properties of the coatings, which will be the focus of the next work by the authors.

## 4. Conclusions

The binary Ti-Cu and ternary Ti-Cu-Ag thin films were deposited by PVD magnetron co-sputtering using a multi-target reactor. The purpose of this study is to understand the influence of Ag on the mechanical and structural properties of the thin films. All the thin films were homogenously thick and very smooth, with a maximum average roughness of 1.2 nm. With regards to the silver content, thin films showed amorphous structure up to 20 at.% of Ag that changed to partially crystalline at 25 at.% and then completely crystalline at higher Ag content in the thin film. The addition of silver content contributed to a significant increase of the thin film adhesion, but reduced both elastic modulus from 124.5 to 105.5 GPa and hardness from 7.8 to 6 GPa. Ti-Cu thin films showed compressive residual stress and changed to completely tensile stresses with the addition of silver in the thin films. Based on the multi-technique characterization, it can be concluded that a suggested Ag composition of about 25 at.% exists to find the optimal mechanical properties of Ti-Cu-Ag sputtered thin films (i.e., improved adhesion and sufficiently good hardness), still maintaining the potential of improving antibacterial properties thanks to silver addition and very small average surface roughness. Further work will focus on the optimization of the Ti-Cu-Ag composition and structure in order to investigate the biocompatibility and antibacterial (or even antiviral) properties of the thin films.

## Figures and Tables

**Table 1:** Power supplied to metallic targets to obtain the desired compositions.

Sample Composition	Ti:Cu ~ 1	Power at Ti (W)	Power at Cu (W)	Power at Ag (W)
Ti-Cu (Control)	48:52	150	29	0
Ti-Cu—10%Ag	43:47	143	27	3
Ti-Cu—15%Ag	40.5:44.5	135	26	4
Ti-Cu—20%Ag	38:42	128	24	5
Ti-Cu—25%Ag	35.5:39.5	120	23	7
Ti-Cu—30%Ag	33:37	113	21	9
Ti-Cu—35%Ag	30.75:34.25	105	20	11

## Data Availability

The data presented in this study are available on request from the corresponding author.
